# Apoptosis of vaginal epithelial cells in clinical samples from women with diagnosed bacterial vaginosis

**DOI:** 10.1038/s41598-020-58862-2

**Published:** 2020-02-06

**Authors:** Elena Roselletti, Samuele Sabbatini, Stefano Perito, Antonella Mencacci, Anna Vecchiarelli, Claudia Monari

**Affiliations:** Department of Medicine, University of Perugia, 06132 - Sant’Andrea delle Fratte, Perugia, Italy

**Keywords:** Bacterial infection, Bacterial infection, Infection, Infection

## Abstract

Bacterial vaginosis (BV) is one of the most common vaginal infections among women of childbearing age*. Gardnerella vaginalis* (*G. vaginalis*) is a keystone microorganism present in more than 95% of all BV cases. The first step of the infection process in BV is mediated by interaction of microorganisms with epithelial cells (ECs). However, the role of these cells in BV pathogenesis is largely unknown. The present study aimed to investigate the vaginal EC response during BV. Twenty healthy women and 34 women with BV were enrolled in this study. The number of ECs in the vaginal swab was counted and analyzed for intracellular signals and apoptosis by flow cytometry. Cell damage was evaluated by lactate dehydrogenase assay. Compared to that in healthy donors, the percentage of exfoliated vaginal ECs was increased in women with BV, and an absence of neutrophils was observed in both groups. Activation signals, such as p-IκBα and c-Fos were unmodulated in the vaginal ECs of women with BV. Moreover, EC damage and apoptosis were significantly increased in patients with BV. Apoptosis was related to caspase-3 activation and the presence of *G. vaginalis*. This study provides the first evidence of a direct involvement of *G. vaginalis* in the apoptotic process of vaginal ECs during BV. This effect was mediated by caspase-3 activation, and *G. vaginalis* appeared to be one of causes for inducing EC apoptosis in BV. Hence, our findings suggest a possible explanation for the increased exfoliation of ECs in the vagina during BV.

## Introduction

Bacterial vaginosis (BV) is one of the most common vaginal infections among women of childbearing age. BV is characterized by a shift in the vaginal microbiota, particularly from the dominant *Lactobacillus* spp. to a subsequent increase in the abundance of anaerobic bacteria, including *Atopobium vaginae (A. vaginae)*, *Prevotella bivia* (*P. bivia*), *Megasphaera*, *Mobiluncus* spp., *Mycoplasma hominis* (*M. hominis*), and *Gardnerella vaginalis* (*G. vaginalis*)^1^.

Dramatic changes in the quantitative and qualitative plethora of bacterial species have also been associated with a broad spectrum of health problems, including pelvic inflammatory disease, preterm births, and increased susceptibility to HIV infection^2^.

*G. vaginalis* remains the best-studied species associated with BV, and was, previously, reported by Gardner and Dukes^3^ to be the sole etiological agent of BV; however, the presence of *G. vaginalis* in healthy women has led to speculation about its virulence potential. In this regard, recent studies showed that some virulence properties of *G. vaginalis* were more highly expressed in isolates from women with BV than in isolates from healthy women^4^. Furthermore, it has been reported that *G. vaginalis* has a significantly higher virulence potential than many other BV-related microorganisms due to its various properties, such as initial adhesion to the epithelium, relatively superior cytotoxicity, and greater ability to form biofilms^5^.

The diagnosis of BV is based on the detection of predominant bacterial vaginosis-associated organisms, particularly *G. vaginalis*, which has the ability to form a dense biofilm adhering to the vaginal epithelium^1^. Biofilm formation is an important virulence factor because it largely contributes to resistance to host immune defense and antibiotic tolerance. Furthermore, *G. vaginalis* forms a significantly thicker biofilm compared to other BV-associated anaerobes^5^. Of note, the biofilms may ascend to the endometrium, causing pelvic inflammatory disease and risk of adverse pregnancy outcome^6^.

BV is, usually, characterized by the absence of neutrophils^7,8^. In an *in vivo* experimental model of vaginal infection with *G. vaginalis*, we demonstrated an absence of vaginal inflammation with a modest induction of anti-inflammatory cytokines, such as IL-10 ^9^. We also demonstrated that *G. vaginalis* efficiently adheres to epithelial cells (ECs)^9^. ECs are the site of initial interaction with a variety of bacteria and/or fungi and may account for different host responses. Recently, we observed that ECs play a key role in inducing inflammatory processes during vaginal candidiasis^10^. However, despite advances in our understanding, BV remains an enigmatic condition in which the role of vaginal ECs is unclear. In this study, we evaluated the status of vaginal ECs in women with BV by assessing the potential relationship between *G. vaginalis* and immune activation.

## Results

**Figure 1:**
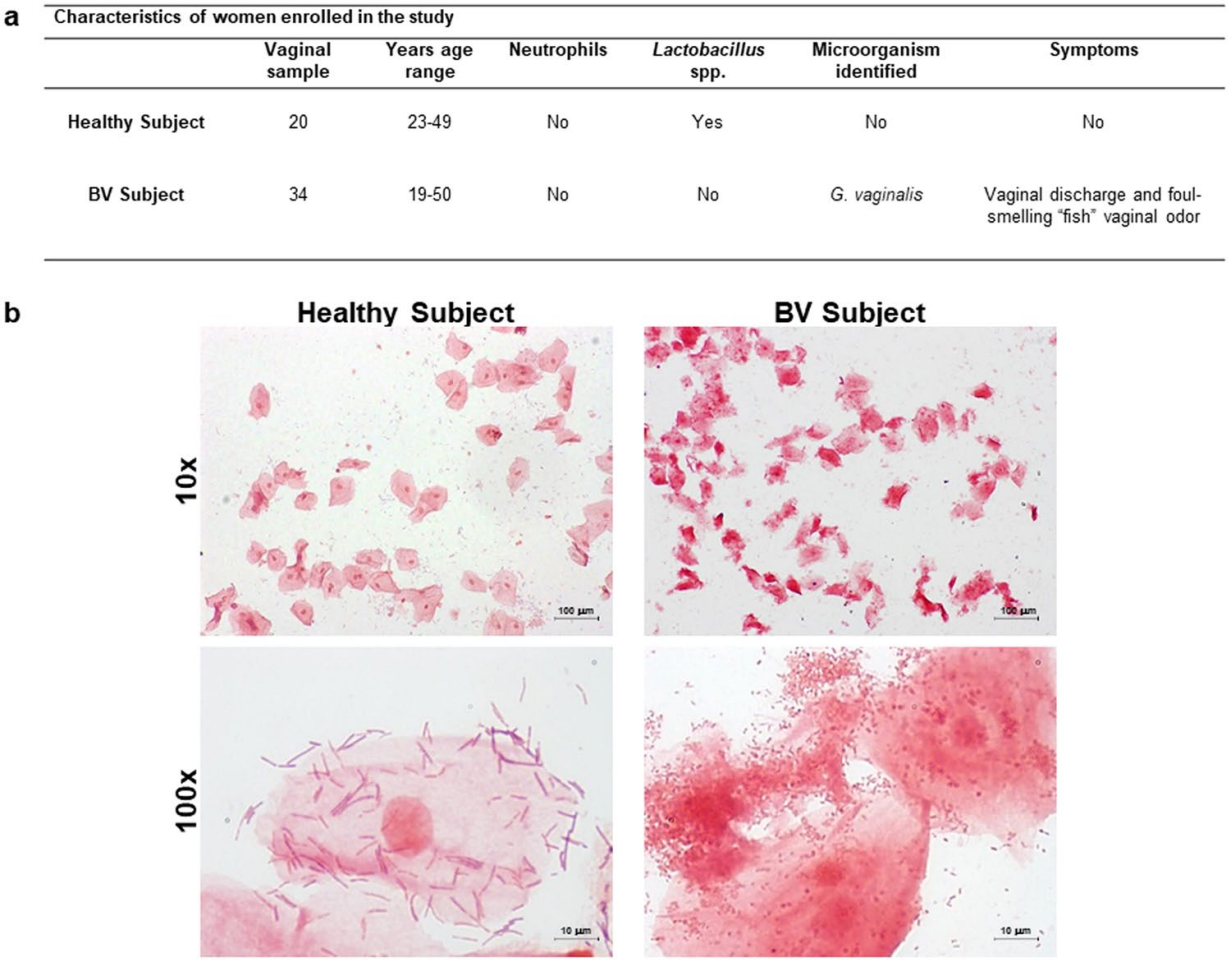
Characteristics of the women enrolled in the present study. The number of patients, age range, the presence of neutrophils, *Lactobacillus* spp. or other microorganisms and symptoms of the vaginal samples obtained from women enrolled in the study are shown (**a**). Vaginal samples from healthy donors (n = 20) and women with BV (n = 34) were examined microscopically to evaluate the presence of lactobacilli or other bacteria following Gram-staining. Representative images of each type of vaginal samples from two different women are shown (original magnification ×100; scale bar: 100 μm and enlarged view of original magnification ×1000, scale bar: 10 μm) (**b**).

It has been previously reported that BV is characterized by decreased abundance of *Lactobacillus* spp. and increased abundance of anaerobic bacteria, particularly *G. vaginalis*^3^. First, we identified *G. vaginalis* isolates by MALDI-TOF MS analysis. Next, we determined the presence of *Lactobacillus* spp., as well as, the number of ECs in our clinical samples. As shown in Fig. 1, we observed the presence of *G. vaginalis* and complete absence of lactobacilli in BV specimens. Conversely, healthy donor samples showed a very low number or the absence of *G. vaginalis* cells and a microbiota dominated by lactobacilli. Furthermore, the complete absence of neutrophils was reported in both groups (Fig. 1).

**Figure 2:**
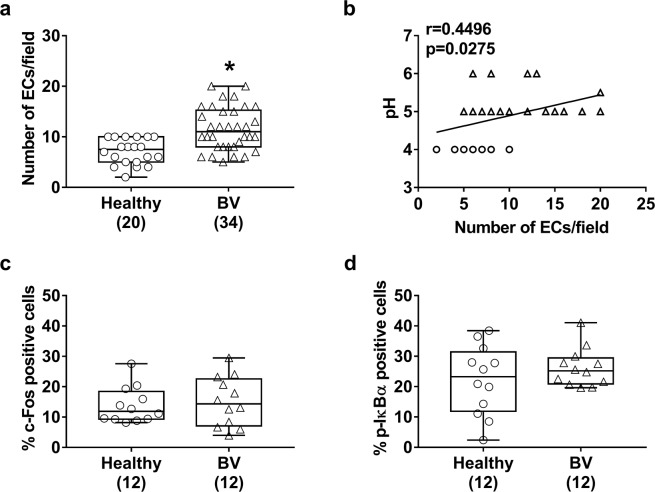
Determination of the number of ECs/field and flow cytometric analysis of c-Fos and p-IκBα in the vaginal samples. Vaginal samples from healthy donors (n = 20) and women with BV (n = 34) were examined under a light microscope to evaluate the number of ECs/field. The statistical significance of differences between the groups was determined with a Mann–Whitney U-test. *P < 0.0001 BV *vs*. healthy women (**a**). Correlation between the number of ECs/field and pH in selected healthy donors (○) and women with BV (Δ) was also evaluated. The linear regression lines are shown. Spearman’s correlation (r) and statistical significance are indicated in the graph. (**b**). The total cellular fractions obtained from selected vaginal swabs of healthy donors (n = 12) and women with BV (n = 12) were labeled with a FITC anti-human c-Fos antibody (**c**) or with FITC anti-human p-IκBα antibody (**d**). A total of 10,000 events were analyzed by flow cytometry. Data are expressed as the percentage positive cells. The boxplot graphics show median including the 25^th^ and 75^th^ percentiles. The statistical significance of differences between the groups was determined with a Mann–Whitney U-test. c–Fos: *P* = 0.8428 BV *vs*. healthy women (**c**); p-IĸBα: *P* = 0.5512 BV *vs*. healthy women (**d**).

In subsequent experiments, we evaluated the amount of EC exfoliation in clinical samples from women with BV. As shown in Fig. 2a, the number of exfoliated ECs increased in the vaginal environment during BV relative to that in healthy donors. It has been reported that the vaginal pH increases during vaginal infections and assessment of vaginal pH may be useful in evaluating vaginal health^11^. Determination of the pH level of our clinical samples showed a pH < 4.5 (4.0 ± 0.0) in healthy women, while a pH, consistently, > 4.5 (5.16 ± 0.06) in patients with BV. In addition we observed a significant correlation between the number of exfoliated ECs and pH values of our specimens (Fig. 2b).

Previous *in vitro* studies have shown enhanced inflammatory response and tissue damage by *G. vaginalis*^5,8^. Indeed, activation of vaginal ECs may occur via the c-Fos/p38 signaling pathway during vaginal candidiasis^10,12^; however, the contribution of these signaling molecules in the downstream molecular cascade in vaginal ECs during bacterial infections remains unknown. Evaluation of c-Fos levels in the vaginal ECs from patients with BV and healthy donors, no difference in levels was observed between the two groups (Fig. 2c).

Several transcription factors, including those related to the IκB kinase/NF-κB signaling pathway, are critically involved in inflammatory response against invading microorganisms^10,13^. Therefore, we analyzed the activation of the NF-κB signaling pathway in vaginal ECs from 12 women with BV and 12 healthy donors. No differences in activation of NF-κB signals were detected between the two groups (Fig. 2d).

**Figure 3:**
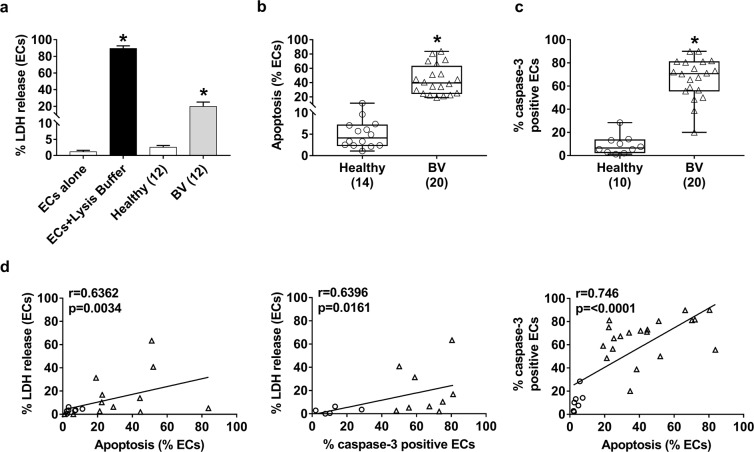
Percentage LDH release in vaginal samples, percentage of vaginal ECs undergoing apoptosis, flow cytometric analysis of active caspase-3 and correlations. Vaginal ECs (1 × 10^6^/mL) from selected healthy donors (n = 12) and women with BV (n = 12) were incubated in PBS for 18 h at 37 °C under 5% CO_2_ in 96-well microtiter plates (100 µL/well). The extent of EC damage was determined by the release of LDH. The graph shown mean ± SEM and statistical significance of differences between the groups was determined with a Kruskal-Wallis test + Dunn’s as a post-hoc comparison. *P < 0.0001 ECs + Lysis Buffer *vs*. ECs alone; *P = 0.0413 BV *vs*. healthy women (**a**). Vaginal ECs from selected healthy donors (n = 14) and women with BV (n = 20) were centrifuged, suspended in hypotonic propidium iodide solution and kept for 1 h in the dark at room temperature. Data are expressed as the percentage of vaginal ECs undergoing apoptosis. The boxplot graphics show median including the 25^th^ and 75^th^ percentiles. The statistical significance of differences between the groups was determined with a Mann–Whitney U test. *P < 0.0001 BV *vs*. healthy women (**b**). At the same time, the total cellular fractions obtained from the same vaginal swabs of healthy donors (n = 10) and women with BV (n = 20) were labeled with PE anti-human active caspase-3 antibody. A total of 10,000 events were analyzed by flow cytometry. Data are expressed as the percentage positive cells. The boxplot graphics show median including the 25^th^ and 75^th^ percentiles. The statistical significance of differences between the groups was determined with a Mann–Whitney U-test. *P < 0.0001 BV *vs*. healthy women (**c**). Correlations between the percentage LDH release *vs*. the percentage vaginal ECs undergoing apoptosis, the percentage LDH release *vs*. caspase-3 positive cells and vaginal ECs undergoing apoptosis *vs*. caspase-3 positive cells in selected healthy donors (○) and women with BV (Δ) were also evaluated. The linear regression lines are shown. A Spearman’s correlation (r) and statistical significance are indicated in the graphs. (**d**).

Previous reports indicate that *G. vaginalis* induces EC damage^5^; therefore, we performed LDH assay to investigate this effect in our samples from healthy donors (n = 12) and patients with BV (n = 12). The results showed a consistent increase in the percentage of damaged vaginal ECs in samples from women with BV compared to those from healthy donors (Fig. 3a).

Next, we assessed LDH release, which may reflect apoptosis^14^, to determine the level of EC apoptosis in clinical samples from women with BV (n = 20) and healthy donors (n = 14). As shown in Fig. 3b, a significant increase in the percentage of apoptotic ECs was detected in samples from patients with BV (43.86% ± 4.53%) compared to those from healthy women.

Caspase-3 is a crucial mediator of apoptosis in both extrinsic (dead ligand) and intrinsic (mitochondrial) pathways^15^; therefore, we evaluated the contribution of caspase-3 in the apoptotic process. As shown in Fig. 3c, consistent activation of caspase-3 was observed in vaginal ECs from women with BV, whereas caspase-3 was very poorly expressed or absent in ECs from healthy donors. A significant correlation between the percentage of LDH release and the percentage of vaginal ECs undergoing apoptosis or the percentage of caspase-3-positive cells was observed in BV and healthy subjects. Furthermore, a positive correlation between caspase-3 levels and EC apoptosis was also observed (Fig. 3d).

**Figure 4:**
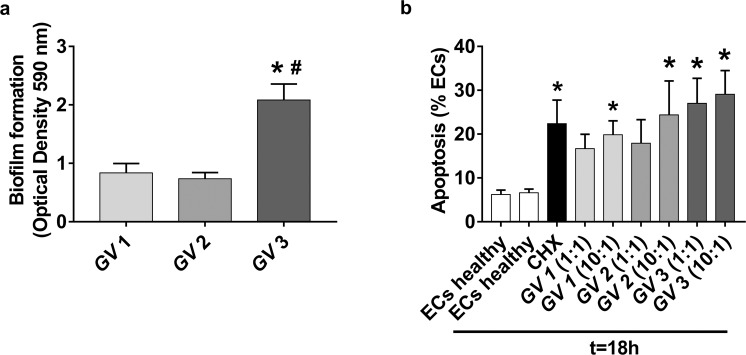
*In vitro* models with *G. vaginalis* clinical isolate. Three *G. vaginalis* clinical isolates (*GV* 1, *GV* 2 and *GV* 3) were obtained from vaginal swabs of subjects enrolled in this study and their ability to form biofilms was evaluated. Biofilm biomass was quantified using the crystal violet (CV) staining method after 48 h of incubation. The optical density (OD) was measured at 590 nm using a 96-well microplates reader. Data represent the mean ± SEM of duplicate samples from three different experiments. The statistical significance of differences between the groups was determined by ANOVA + Bonferroni as a post-hoc comparison. *P = 0.0111 *GV* 3 *vs*. *GV* 1; ^#^*P* = 0.0077 *GV* 3 *vs. GV* 2; *P* > 0.9999 *GV* 1 *vs. GV* 2 (**a**). Vaginal ECs from healthy donors (n = 9) were washed three times, counted, adjusted to 2×10^5^/ml in RPMI 1640 supplemented with 10% FCS. Cells were then cultured for 18 h at 37 °C under 5% CO_2_ in 96-well microtiter plates (100 µl/well) with or without cycloheximide (CHX) 100 μg/ml, *GV* 1, *GV* 2 and *GV* 3 at 2×10^5^/ml, 100 µl/well (1:1) or *GV* 1, *GV* 2 and *GV* 3 at 2×10^6^/ml, 100 µl/well (10:1). Immediately (t = 0) and after incubation, ECs were centrifuged, suspended in hypotonic propidium iodide solution and kept for 1 h in the dark at room temperature. Data are expressed as the percentage of vaginal ECs undergoing apoptosis. Data represent the mean ± SEM of duplicate samples from 9 different women for *GV3* and 5 different women for *GV 1* and *GV 2*. The statistical significance of differences between the groups was determined by Kruskal-Wallis + Dunn’s as a post-hoc comparison. *P = 0.0025 CHX *vs*. ECs from healthy donors (t = 18 h); *P = 0.0119 *GV* 1 (10:1) *vs*. ECs from healthy donors (t = 18 h); *P = 0.0175 *GV* 2 (10:1) *vs*. ECs from healthy donors (t = 18 h); *P = 0.0012 *GV* 3 (1:1) *vs*. ECs from healthy donors (t = 18 h); *P = 0.0002 *GV* 3 (10:1) *vs*. ECs from healthy donors (t = 18 h); *P* > 0.9999 ECs from healthy donors (t = 0) *vs*. ECs from healthy donors (t = 18 h); *P* = 0.1779 *GV* 1 (1:1) *vs*. ECs from healthy donors (t = 18 h); *P* = 0.1692 *GV* 2 (1:1) *vs*. ECs from healthy donors (t = 18 h) (**b**).

To determine the relationship between EC apoptosis and the presence of *G. vaginalis* cells, we performed *ex vivo* experiments using the vaginal ECs of selected healthy subjects that were treated with *G. vaginalis* clinical isolates from three women enrolled in this study. Given that BV can be considered a biofilm infection primarily consisting of *G. vaginalis*^16^, we evaluated the biofilm-forming ability of the three *G. vaginalis* clinical isolates (designated *GV* 1, *GV* 2, and *GV* 3) under our experimental conditions. To this end, the strains (10^8^ CFU/ml) were incubated for 48 h and biofilm formation on an abiotic surface (polystyrene plates) was analyzed by CV staining, as described in the Methods section. As shown in Fig. 4a, *GV* 3 strain exhibited the highest biofilm-forming ability. In subsequent experiments, the ability of strains *GV* 1, *GV* 2, and *GV* 3 to induce vaginal EC apoptosis in healthy subjects was evaluated. As shown in Fig. 4b, *GV* 3 induced vaginal EC apoptosis at all concentrations tested, while *GV* 1 and *GV* 2 were able to induce apoptosis of vaginal ECs only at the highest concentration.

## Discussion

BV is one of the most common pathological conditions among women of childbearing age. Vaginal dysbiosis and BV usually occur when *Lactobacillus* spp., which is the predominant species in the vagina of healthy women, is replaced by various anaerobic bacteria, particularly *G. vaginalis*. Despite the relevance of this infection and its impact on public health, BV remains an ambiguous condition.

In this study, we demonstrated the dysregulation of a series of parameters in samples from women with BV. Our results showed: 1) a consistent increase in pH level, 2) an increased number of exfoliated ECs, 3) a lack of certain inflammatory signals, 4) increased vaginal EC damage, and 5) increased vaginal EC apoptosis via caspase-3 activation. Finally, even though we cannot exclude the involvement of other bacteria, our data demonstrate that *G. vaginalis* plays an important role in inducing apoptosis of vaginal ECs.

Compelling evidence shows that *Lactobacillus* spp. produce lactic acid and peroxide hydrogen, which contribute to a hostile environment for the growth of harmful organisms^17^. BV implies a modification of the vaginal microbiota, with increased colonization of several anaerobic or facultative microorganisms and a consequent increase in the pH level^18^. Compared to the samples from healthy subjects, in the BV samples, we observed an increase in pH value, as well as, an augmentation of ECs exfoliation. This was due to an increase in the population of anaerobic bacteria in the vagina and, particularly, to the presence of *G. vaginalis*. Hence, it can be speculated that the production of vaginolysin (VGL), which is the most important virulence factor of *G. vaginalis*, is responsible for the enhanced exfoliation^19^. Although we were unable to assess the VGL levels of the strains in our study, previous studies have suggested that higher VGL concentrations are related to BV with rare lactobacilli in the vaginal microbiota^20^, a condition that was also manifested in our clinical samples. Furthermore, we identified a positive relationship between increased pH and augmented exfoliation.

ECs are in constant contact with various microbes and respond to pathogenic organisms. This condition elicits an appropriate immune response via activation of cellular signalling pathways, such as c-Fos and NF-κB, similar to that induced by fungal infections^10^. In our samples, we did not observe the activation of selected inflammatory signaling molecules in vaginal ECs. However, we cannot exclude that, during BV, there is activation of alternative intracellular signals that leads to production of inflammatory cytokines. To our knowledge, this is the first study to demonstrate that vaginal ECs are unresponsive to microorganisms during BV. Furthermore, we also demonstrated that, consistent vaginal ECs damage was manifested. Indeed, previously published data were obtained exclusively from *in vitro* experimental models using epithelial cell lines^5,21^.

In this study, we provide evidence of vaginal EC apoptosis during BV via a process that involves caspase-3 activation. Given that vaginal ECs are strongly involved in BV as clue cells, it is likely that the enhanced apoptosis represents host response, which removes the infected and exhausted cells. The absence of NF-κB activation correlates with the increased apoptosis observed in ECs from the intestinal region^22,23^. Our findings, also, reveal that the presence of *G. vaginalis* is strictly associated with apoptosis induction, although we cannot exclude the possible contribution of other factors and microorganisms to this phenomenon.

Apoptosis in response to invading pathogens may represent a host defense mechanism for the elimination of infected cells and/or a strategy by which microorganisms disturb the EC barrier against invasion of deeper mucosal layers required for prolonged bacterial colonization. In this study, we provide evidence that *G. vaginalis* induces apoptosis of vaginal ECs, although the underlying mechanism remains unknown. In addition, the presence of other microorganisms in the vaginal environment should be considered in future studies.

Even though the apoptotic process of ECs has been previously described in BV^24,25^, this study provides evidence of a direct involvement of *G. vaginalis* in vaginal apoptosis of epithelial cells during BV. The existence of a cell death pathway regulated by *G. vaginalis* provides a possible explanation for increased vaginal EC exfoliation and highlights the potential of pharmacological manipulation of EC apoptosis as a novel strategy for treating BV.

## Methods

### Participants

A total of 54 Caucasian, non-pregnant, non-menopausal, and non-diabetic women (aged 19–50 years) attending the microbiological diagnostic service at the University Hospital Santa Maria della Misericordia, Perugia (Italy) from November 2018 to July 2019 were randomly enrolled in this study. The exclusion criteria were: patients outside the age range of 19–50 years, patients with a history of tubal ligation or hysterectomy, and women with *Chlamydia trachomatis*, *Neisseria gonorrhoeae*, *Trichomonas vaginalis*, *Streptococcus agalactiae*, *Saccharomyces cerevisiae, Candida* spp., or HIV infections^19^. Individuals were excluded from the study if they had received systemic or local antibiotic/antimicrobial therapy within the previous 2 months, were menstruating at the time of examination, were using an intrauterine contraceptive device, or had a history of sexually transmitted diseases^1^. The subjects enrolled in this study were divided into two groups: 34 women with BV and 20 healthy women (Fig. 1a). Diagnosis of BV was based on Amsel’s and Nugent’s criteria^1,8,19,26^. Prior to enrollment, all patients completed a questionnaire indicating their health status and symptoms of vaginal disease. All participants provided written informed consent in accordance with the Declaration of Helsinki. Local Ethical Committee CEAS (Comitato Etico delle Aziende Sanitarie, Umbria, Italy) approval was received for the entire study (MICROCA). All methods were performed in accordance with the relevant guidelines and regulations.

### Sample collection

Three vaginal swabs were collected from each woman enrolled in this study. One swab was used for pH measurement using pH-Fix strips (Macherey-Nagel GmbH & Co. KG, Düren, Germany).

One swab was soaked in 1 ml of saline. A certain volume of this sample was cultured on BD Columbia CNA Agar containing 5% Sheep Blood (Becton Dickinson, New Jersey, USA). For *G. vaginalis* identification, mass-spectrometry (MALDI-TOF MS) analysis (Bruker Daltonik GmbH, Bremen, Germany) was performed. The remaining volume of the sample was used for Gram stain (Nugent’s criteria) for the diagnosis of BV. The last swab was soaked in 1 ml of saline and vortexed for at least one minute. Around 100 μL of the sample was examined under a light microscope (Olympus, Milan, Italy) to evaluate the presence of neutrophils (PMNs) and EC exfoliation. The numbers of PMNs and ECs were counted in four fields at ×400 magnification and expressed as the average number of PMNs or ECs/field, as previously described^10,19,27^.

The remaining sample (900 μl) was centrifuged at 1,600 rpm for 10 min, and the cellular fraction was used for flow cytometric analysis or for assessment of EC damage or EC apoptosis.

Due to the limited amount of ECs in our samples, we were unable to perform all analyses for all samples. In each figure, we have reported the number of BV and healthy specimens used.

### MALDI-TOF MS

For MALDI-TOF MS, bacteria were grown in the swab culture, as described above, and bacterial extracts were prepared as described previously^28,29^. Briefly, cells from a single colony of fresh vaginal sample culture were used to prepare samples according to the microorganism profiling ethanol/acid formic extraction procedure, as per the manufacturer’s instructions. After centrifugation at maximum speed for 2 min, 1 μl of the supernatant containing the bacterial extract was allowed to dry after overlaying it with 2 μl of a chemical matrix (saturated solution of α-cyano-4-hydroxy-cinnamic acid in 50% acetonitrile/2.5% trifluoroacetic acid) on a polished steel MALDI target plate. The samples were then processed using the microflex LT mass spectrometer (Bruker Daltonik GmbH, Bremen, Germany) equipped with a 20-Hz nitrogen laser. The spectra were recorded in the positive linear mode as described previously^30^.

### Flow cytometric analysis

The total cellular fractions obtained from selected vaginal swabs were fixed in 1.5% formalin for 5 min at room temperature, washed, and analyzed by flow cytometry (FACSCalibur, BD Biosciences, New Jersey, USA) to determine FSC/SSC vaginal EC parameter, as previously reported^10,31^, or incubated for 20 min at room temperature in the dark with a FITC-conjugated mouse monoclonal antibody to human epithelial cell adhesion molecule (EpCAM, CD326) (IgG2_ak_) (0.5 µg/test; eBioscience, Thermo Fisher Scientific, California, USA), or permeabilized with absolute methanol (500 µl/10^6^ cells) for 10 min on ice and then incubated with a FITC-conjugated mouse monoclonal antibody to human p-IκBα (IgG_2bk_) or human c-Fos (IgG_1k_) (both dilution 1:50; Santa Cruz Biotechnology, California, USA) or PE-conjugated rabbit monoclonal antibody to human active caspase-3 (IgG) (5 μl/test, BD Pharmingen^TM^, Allschwil, Switzerland). After incubation, the cells were washed with Fluorescein Buffer and 10,000 events were analyzed by flow cytometry. More than 98% of the cells contained within the gate were EpCAM-positive. Isotype controls, including mouse IgG_2ak_, IgG_2bk_, and IgG_1k_ and rabbit IgG were purchased from Novus Biologicals (Colorado, USA). PE-conjugated sheep anti-mouse IgG (whole molecule) and FITC-conjugated mouse anti-rabbit IgG (whole molecule) were purchased from Sigma-Aldrich (Munich, Germany) and Santa Cruz Biotechnology (California, USA), respectively. Autofluorescence was assessed using untreated cells. Specific fluorescence was assessed by comparison with results from the specific mouse isotype control. The data were expressed as the percentage of positive cells.

### EC damage assay

The number of ECs in the selected vaginal samples was counted, adjusted to 1 × 10^6^/ml in phosphate-buffered saline (PBS), and cultured for 18 h at 37 °C under 5% CO_2_ in 96-well microtiter plates (100 µl/well). Subsequently, EC damage was determined by the release of lactate dehydrogenase (LDH) into the surrounding medium using the Pierce LDH Cytotoxicity Detection kit (Thermo Fisher Scientific, California, USA). For each test, ECs from one healthy donor were used as the negative control (ECs cultured in PBS alone for spontaneous LDH activity) and positive control (ECs + lysis buffer in PBS for maximum LDH activity). LDH activity was measured spectrophotometrically at 492 nm. The percentage of damaged vaginal ECs was calculated as follows: (LDH activity of vaginal cells − spontaneous LDH activity/maximum LDH activity − spontaneous LDH activity) × 100^32^.

### Evaluation of apoptosis by propidium iodide staining

The percentage of vaginal ECs undergoing apoptosis was quantified by staining with propidium iodide (PI) (50 μg/ml; Sigma-Aldrich, Munich, Germany) according to the manufacturer’s instructions. Briefly, vaginal ECs were centrifuged at 1,600 rpm for 10 min, suspended in hypotonic PI solution, and kept for 1 h in the dark at room temperature. The PI fluorescence of individual nuclei was measured by flow cytometry and the percentage of apoptotic vaginal EC nuclei was calculated using FACScan research software (BD Biosciences, New Jersey, USA) as previously described^33,34^.

### Microbial strains and growth conditions

*G. vaginalis* clinical isolates were obtained from the vaginal swabs of 3 women with BV enrolled in this study at the Microbiology Department of Santa Maria della Misericordia Hospital of Perugia. These swabs were used to inoculate Gardnerella selective agar (GSA) plates containing 5% human blood (Becton Dickinson, New Jersey, USA). Plates were incubated anerobically at 37 °C for 24–48 h, β-hemolytic colonies were isolated, and candidate *G. vaginalis* strains were identified by mass-spectrometry (MALDI-TOF MS). *G. vaginalis* clinical isolates were then cultured in Brain Heart Infusion (BHI) broth and incubated anaerobically at 37 °C.

### Biofilm formation

Biofilms were grown using 96-well plates. Overnight cultures of three *G. vaginalis* clinical isolates obtained from women with BV were adjusted to a concentration of 1×10^8^ CFU/ml in sBHI (BHI broth supplemented with 2% gelatin, 1% yeast extract, 0.1% soluble starch, and 0.25% maltose), and samples (100 μl) were transferred to each well of the plate. Plates were then incubated for 48 h at 37 °C in 10% CO_2_. To evaluate the effect of fed-batch growth on biofilm formation (48 h), the culture medium was replaced with fresh medium after 24 h of growth.

### Biofilm quantification

Biofilm biomass was quantified using crystal violet (CV) staining as previously described^35,36^. CV is a basic dye, which binds to negatively charged surface molecules and polysaccharides in the extracellular matrix. After biofilm formation (48 h), the biofilm was washed twice with 200 μl PBS and fixed with 100 μl methanol. After 15 min, the supernatants were discarded and the plate was air-dried. The biofilms were then stained with 100 μl 0.5% CV for 20 min. Subsequently, the plates were washed twice with 200 μl PBS to remove excess of CV. Finally, CV was solubilized by adding 150 μl of 33% acetic acid per well. The optical density (OD) at 590 nm was measured using a 96-well microplate reader (Tecan, Männedorf, Switzerland).

### Evaluation of apoptosis by PI staining in an *ex vivo* model

Vaginal samples from 9 healthy women (BV-free) were washed three times with PBS at 1,000 rpm for 5 min to eliminate most commensal bacteria (*Lactobacillus* spp.). The vaginal ECs were then counted and their concentration was adjusted to 2×10^5^/ml in RPMI 1640 medium supplemented with 10% fetal calf serum (FCS). Cells were cultured for 18 h at 37 °C under 5% CO_2_ in 96-well microtiter plates (100 µl/well) with or without cycloheximide (CHX; 100 μg/ml)^37^ or *G. vaginalis* clinical isolate (2×10^5^/ml or 2×10^6^/ml in BHI broth, 100 µl/well). The percentage of vaginal ECs undergoing apoptosis was quantified at time 0 and 18 h after staining with PI, as described previously^33,34^. During the incubation period, antibiotics were not added to the medium.

### Statistical analysis

Data were presented as the mean ± standard error of the mean (SEM) from triplicate samples or as boxplot graphics with median and the 25^th^ and 75^th^ percentile in which all women were identified by dots. GraphPad Prism 7.0 software was used for statistical analyses and to test for normal distribution. Non-parametric Spearman’s correlation test was used to assess the correlation between two variables. Data were analyzed by ANOVA followed by Bonferroni post-hoc test, or Kruskal-Wallis test followed by Dunn’s post-hoc comparison test, or Mann–Whitney U test, as indicated in each figure. Values of *P* < 0.05 were considered to be statistically significant.

## Data Availability

The data supporting the conclusions of this manuscript will be made available by the corresponding author upon reasonable request.

## References

[1] Swidsinski A (2005). Adherent biofilms in bacterial vaginosis. Obstet Gynecol.

[2] Schwebke JR, Muzny CA, Josey WE (2014). Role of Gardnerella vaginalis in the pathogenesis of bacterial vaginosis: a conceptual model. J Infect Dis.

[3] Gardner HL, Dukes CD (1959). Hemophilus vaginalis vaginitis. Ann N Y Acad Sci.

[4] Castro J, Machado D, Cerca N (2019). Unveiling the role of Gardnerella vaginalis in polymicrobial Bacterial Vaginosis biofilms: the impact of other vaginal pathogens living as neighbors. ISME J.

[5] Patterson JL, Stull-Lane A, Girerd PH, Jefferson KK (2010). Analysis of adherence, biofilm formation and cytotoxicity suggests a greater virulence potential of Gardnerella vaginalis relative to other bacterial-vaginosis-associated anaerobes. Microbiology.

[6] Swidsinski A (2013). Presence of a polymicrobial endometrial biofilm in patients with bacterial vaginosis. PLoS One.

[7] Kalia N, Singh J, Kaur M (2019). Immunopathology of Recurrent Vulvovaginal Infections: New Aspects and Research Directions. Front Immunol.

[8] Onderdonk AB, Delaney ML, Fichorova RN (2016). The Human Microbiome during Bacterial Vaginosis. Clin Microbiol Rev.

[9] Sabbatini S (2018). Saccharomyces cerevisiae-based probiotic as novel anti-microbial agent for therapy of bacterial vaginosis. Virulence.

[10] Roselletti Elena, Perito Stefano, Sabbatini Samuele, Monari Claudia, Vecchiarelli Anna (2019). Vaginal Epithelial Cells Discriminate Between Yeast and Hyphae of Candida albicans in Women Who Are Colonized or Have Vaginal Candidiasis. The Journal of Infectious Diseases.

[11] Hemalatha R, Ramalaxmi BA, Swetha E, Balakrishna N, Mastromarino P (2013). Evaluation of vaginal pH for detection of bacterial vaginosis. Indian J Med Res.

[12] Nikou, Kichik, Brown, Ponde, Ho, Naglik, Richardson (2019). Candida albicans Interactions with Mucosal Surfaces during Health and Disease. Pathogens.

[13] Akira S, Uematsu S, Takeuchi O (2006). Pathogen recognition and innate immunity. Cell.

[14] Mehta R (2014). Lactate dehydrogenase and caspase activity in nasopharyngeal secretions are predictors of bronchiolitis severity. Influenza Other Respir Viruses.

[15] Elmore S (2007). Apoptosis: a review of programmed cell death. Toxicol Pathol.

[16] Hardy L (2015). Unravelling the Bacterial Vaginosis-Associated Biofilm: A Multiplex Gardnerella vaginalis and Atopobium vaginae Fluorescence *In Situ* Hybridization Assay Using Peptide Nucleic Acid Probes. PLoS One.

[17] Antonio MA, Hawes SE, Hillier SL (1999). The identification of vaginal Lactobacillus species and the demographic and microbiologic characteristics of women colonized by these species. J Infect Dis.

[18] Krauss-Silva L (2014). Basic vaginal pH, bacterial vaginosis and aerobic vaginitis: prevalence in early pregnancy and risk of spontaneous preterm delivery, a prospective study in a low socioeconomic and multiethnic South American population. BMC Pregnancy Childbirth.

[19] Amegashie CP (2017). Relationship between nugent score and vaginal epithelial exfoliation. PLoS One.

[20] Gelber SE, Aguilar JL, Lewis KL, Ratner AJ (2008). Functional and phylogenetic characterization of Vaginolysin, the human-specific cytolysin from Gardnerella vaginalis. J Bacteriol.

[21] Castro J, Martins AP, Rodrigues ME, Cerca N (2018). Lactobacillus crispatus represses vaginolysin expression by BV associated Gardnerella vaginalis and reduces cell cytotoxicity. Anaerobe.

[22] Yan SR (2005). Activation of NF-kappaB following detachment delays apoptosis in intestinal epithelial cells. Oncogene.

[23] Hausmann M (2010). How bacteria-induced apoptosis of intestinal epithelial cells contributes to mucosal inflammation. Int J Inflam.

[24] Ma X, Deng J, Cui X, Chen Q, Wang W (2019). Berberine exhibits antioxidative effects and reduces apoptosis of the vaginal epithelium in bacterial vaginosis. Exp Ther Med.

[25] Chen Z, Zhang Z, Zhang H, Xie B (2015). Analysis of the Oxidative Stress Status in Nonspecific Vaginitis and Its Role in Vaginal Epithelial Cells Apoptosis. Biomed Res Int.

[26] Nugent RP, Krohn MA, Hillier SL (1991). Reliability of diagnosing bacterial vaginosis is improved by a standardized method of gram stain interpretation. J Clin Microbiol.

[27] Roselletti E (2017). NLRP3 inflammasome is a key player in human vulvovaginal disease caused by Candida albicans. Sci Rep.

[28] Mencacci A (2013). Typing of nosocomial outbreaks of Acinetobacter baumannii by use of matrix-assisted laser desorption ionization-time of flight mass spectrometry. J Clin Microbiol.

[29] Spanu T (2011). Evaluation of matrix-assisted laser desorption ionization-time-of-flight mass spectrometry in comparison to rpoB gene sequencing for species identification of bloodstream infection staphylococcal isolates. Clin Microbiol Infect.

[30] Carbonnelle E (2007). Rapid identification of Staphylococci isolated in clinical microbiology laboratories by matrix-assisted laser desorption ionization-time of flight mass spectrometry. J Clin Microbiol.

[31] Hollmer C, Essmann M, Ault K, Larsen B (2006). Adherence and blocking of Candida albicans to cultured vaginal epithelial cells: treatments to decrease adherence. Infect Dis Obstet Gynecol.

[32] Pericolini E (2017). Therapeutic activity of a Saccharomyces cerevisiae-based probiotic and inactivated whole yeast on vaginal candidiasis. Virulence.

[33] Pericolini E (2006). Cryptococcus neoformans capsular polysaccharide component galactoxylomannan induces apoptosis of human T-cells through activation of caspase-8. Cell Microbiol.

[34] Migliorati G, Nicoletti I, Pagliacci MC, D’Adamio L, Riccardi C (1993). Interleukin-4 protects double-negative and CD4 single-positive thymocytes from dexamethasone-induced apoptosis. Blood.

[35] Machado D, Palmeira-de-Oliveira A, Cerca N (2015). Optimization of culture conditions for Gardnerella vaginalis biofilm formation. J Microbiol Methods.

[36] Peeters E, Nelis HJ, Coenye T (2008). Comparison of multiple methods for quantification of microbial biofilms grown in microtiter plates. J Microbiol Methods.

[37] Tang D, Lahti JM, Grenet J, Kidd VJ (1999). Cycloheximide-induced T-cell death is mediated by a Fas-associated death domain-dependent mechanism. J Biol Chem.

